# Supplementary material for: Asynchronous magnetic resonance elastography: Shear wave speed reconstruction using noise correlation of incoherent waves
Source: Magn Reson Med. 2022 Oct 27;89(3):990–1001. doi: 10.1002/mrm.29502 (PMC9792433; doi:10.1002/mrm.29502)
Supplement: Supplementary file 1 — DATA S1 MATLAB code used to generate the numerical simulations of Figure 1 [file MRM-89-990-s002.zip › k-Wave/helpfiles/example_na_filtering.html]

Filtering A Delta Function Input Signal Example (k-Wave)


# Filtering A Delta Function Input Signal Example

This example illustrates how filtering and smoothing can be used to reduce numerical aliasing from delta function and other broadband inputs. The accompanying example MATLAB file is divided into three parts.

## Contents

- Truncated frequency content
- Smoothing the source mask
- Filtering the input time series

## Truncated frequency content

- Open the file in the MATLAB Editor
- Run the file in MATLAB

When a short temporal pressure pulse is applied in a k-Wave simulation, the frequency content of the pulse will typically exceed that supported by the simulation grid. This is because the maximum temporal frequency component of the input signal will be larger than the equivalent maximum spatial frequency component supported by the simulation grid. As the grid cannot propagate these higher frequencies, the frequency content is truncated which distorts the input signal. This can be clearly illustrated in one-dimension by applying a temporal delta function pressure pulse at a single position after some time delay. The figure below shows an input delta pulse and the temporal signal recorded at a sensor position some distance away. By examining the amplitude spectrum of the two signals, it is clear that the frequency content of the pressure field that propagates through the medium is truncated to the maximum frequency supported by the simulation grid. This distorts the propagating wave field.


## Smoothing the source mask

- Open the file in the MATLAB Editor
- Run the file in MATLAB

When running a photoacoustic simulation (where an initial pressure is defined), k-Wave avoids oscillations by spatially smoothing the source mask (see the Source Smoothing Example). The smoothing function rolls off gradually from DC to the maximum frequency supported by the grid. This approach can also be applied when using a simple time varying pressure input by smoothing the source mask and using a scaled version of the input signal according to the values in the source mask. The recorded temporal signal and its amplitude spectrum are shown below (compare these with the time series shown in the preceding example).


## Filtering the input time series

- Open the file in the MATLAB Editor
- Run the file in MATLAB

More control over the frequency content of the input signal can be obtained by temporally filtering the input time series (rather than spatially smoothing the source as in the previous example). This can be achieved using `filterTimeSeries`. By default, this uses a causal FIR filter designed using the Kaiser windowing method (set `example_number = 1` within the example m-file). The causal nature of the filter means that temporal signals will become offset from their original position.

```
% filter the input signal
source_func_filtered = filterTimeSeries(kgrid, medium, source_func);
```

If preserving the temporal location of the input peaks is important, a zero phase filter (non-causal) can be used by setting the option input parameter `'ZeroPhase'` to `true` (set `example_number = 2` within the example m-file). When using a zero phase filter with input signals that vary from the first sample (t = 0), a short ramp should be applied by setting the optional input `'RampPPW'` to `true` to smooth out any start-up transients.

```
% filter the input signal
source_func_filtered = filterTimeSeries(kgrid, medium, source_func, 'ZeroPhase', true);
```

The properties of the filter can be adjusted using optional input parameters. The filter cutoff is adjusting using `'PPW'` which sets the minimum number of grid points per wavelength (PPW) that should be used to propagate the highest frequency component (this is set to 3 by default). The filter transition is adjusted using `'TransitionWidth'` which defines the size of the transition based on the temporal sampling frequency (the default is 0.1). Using a shorter filter transition will cause a larger temporal offset and more more closely preserve the lower frequency content of the input signal (set `example_number = 3` within the example m-file).

```
% filter the input signal
source_func_filtered = filterTimeSeries(kgrid, medium, source_func, 'PPW', 4, 'TransitionWidth', 0.05);
```

The temporal signal recorded using the filtered input signal shown (set `example_number = 3` within the example m-file) above is illustrated below. The input and output signals and their amplitude spectra are almost identical.
